# Biological Role of Gellan Gum in Improving Scaffold Drug Delivery, Cell Adhesion Properties for Tissue Engineering Applications

**DOI:** 10.3390/molecules24244514

**Published:** 2019-12-10

**Authors:** Thangavelu Muthukumar, Jeong Eun Song, Gilson Khang

**Affiliations:** Department of BIN Convergence Technology, Department of Polymer Nano Science & Technology and Polymer BIN Research Center, Chonbuk National University, Deokjin-gu, Jeonju 561-756, Korea; auromuthu@gmail.com (T.M.); songje@jbnu.ac.kr (J.E.S.)

**Keywords:** gellan gum, cell adhesion, tissue engineering, drug delivery, hydrogel, scaffold

## Abstract

Over the past few decades, gellan gum (GG) has attracted substantial research interest in several fields including biomedical and clinical applications. The GG has highly versatile properties like easy bio-fabrication, tunable mechanical, cell adhesion, biocompatibility, biodegradability, drug delivery, and is easy to functionalize. These properties have put forth GG as a promising material in tissue engineering and regenerative medicine fields. Nevertheless, GG alone has poor mechanical strength, stability, and a high gelling temperature in physiological conditions. However, GG physiochemical properties can be enhanced by blending them with other polymers like chitosan, agar, sodium alginate, starch, cellulose, pullulan, polyvinyl chloride, xanthan gum, and other nanomaterials, like gold, silver, or composites. In this review article, we discuss the comprehensive overview and different strategies for the preparation of GG based biomaterial, hydrogels, and scaffolds for drug delivery, wound healing, antimicrobial activity, and cell adhesion. In addition, we have given special attention to tissue engineering applications of GG, which can be combined with another natural, synthetic polymers and nanoparticles, and other composites materials. Overall, this review article clearly presents a summary of the recent advances in research studies on GG for different biomedical applications.

## 1. Introduction

**Figure 1:**
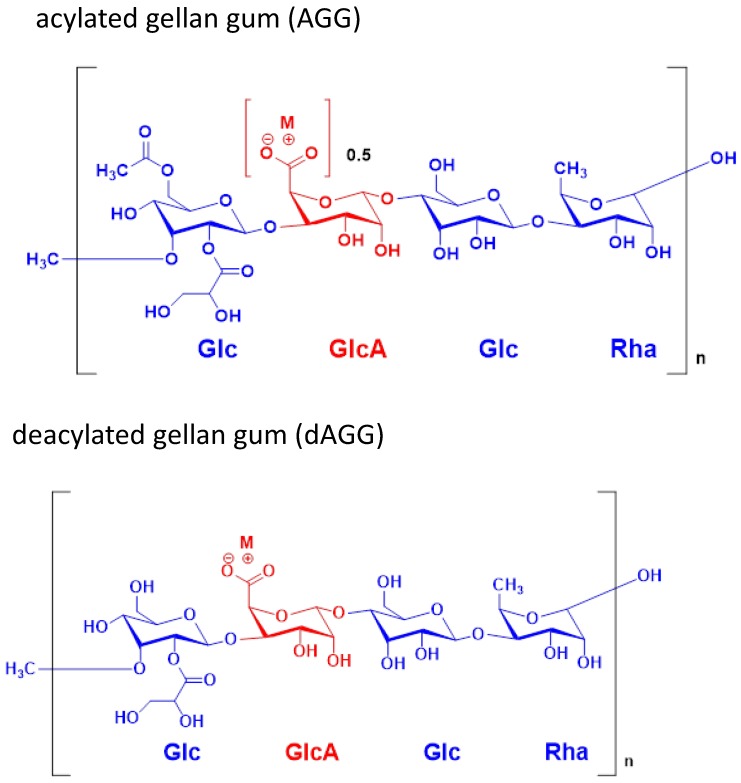
Showing the structure of acylated gellan gum (AGG) and deacylated gellan gum (dAGG).

Gellan gum (GG) is a linear anionic high molecular weight exopolysaccharide, commercially produced by microbial fermentation of the *Sphingomonas paucimobilis* microorganism [1], comprised of tetrasaccharide (1,3-β-d-glucose (Glc), 1,4-β-d-glucuronic acid (GlcA), 1,4-β-d-glucose (Glc), and 1,4-α-l-rhamnose (Rha)) repeating units with one carboxyl side group [2]. Figure 1 shows that GG consist of repeating tetramers of l-rhamnose, d-glucuronic acid, and two d-glucose subunits. GG also contains glycated and acetate functionalities. Deacylated GG is the most commonly used in the tissue engineering (TE) and pharmaceutical fields [3], because of their relative ease of isolation and processing methodology. The GG average molecular weight is about 500 kDa [4]. GG is commercially available under the trade name Gelrite^TM^ (acyl GG or acylated GG) and Kelcogel^TM^ (low acyl GG or deacylated GG) [5,6,7,8]. Other products related to GG can also be found under the trade name of Grovgel, nanogel-TC, Phytagel^®^, and AppliedGel.

GG is thermo-responsive [9], biocompatible [10,11,12,13], nontoxic [8,14,15], ductile [8,16,17], and has the ability to tolerate heat and acid stress during the material fabrication process [18]. The GG composites produce elastic and soft gels, whereas pure GG produces a hard, transparent gel, with rigid and thermally stable products [19]. It has been reported that self-supporting hydrogels of GG can be formed by simple crosslinking with standard cell culturing media, with no added ions [20]. Chemical or covalent crosslinking using a chemical crosslinker, like 1-ethyl-3-(3-dimethylaminopropyl) arbodiimide (EDC), has also been reported for GG gelation [21]. GG gels form as a result of association between double-helical stretches that form ordered junction domains, interconnected by unordered chain segments [22].

**Figure 2:**
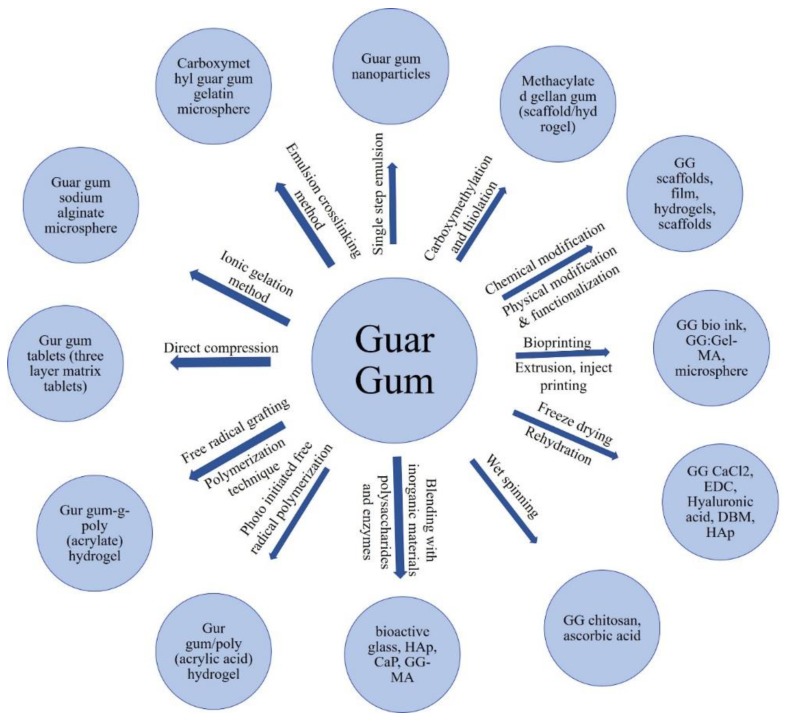
Major routes for preparing gellan gum-based biomaterials [4,32,34,35,36,37,38,39,65,66,67,68,69].

The versatile properties of GG help in different TE and regenerative medicine (RM) applications [23]. The application of GG in cellular and acellular strategies has been successfully suggested for cartilage [24], drug delivery [25,26,27], and intervertebral disc repair [28,29,30]. The major attractive properties of GG that make them a suitable material for TE include its non-cytotoxicity, biocompatibility, structural similarities with native glycosaminoglycans, mild processing conditions, and mechanical properties similar to the elastic moduli of common tissue. The mechanical properties of the GG are improved by combining it with inorganic materials (for their flexibility), and biopolymers (with poor rigidity) became common and smart solutions to improve the mechanical properties of GG. Composites of GG have been recently accomplished by the introduction of hydroxyapatite (HAp) [31], bioactive glass [32,33], calcium phosphate (CaP) [34], hyaluronic acid (HA) [35], demineralized bone powder (DBP) [36], polyethylene glycol [37], silk fibrin [38], agar [39], saponin [40], and chondroitin sulfate [41]. Table 1 shows the different types of GG composites that are used for biological applications. GG is used in the pharmaceutical and biomedical fields, including for gene transfection, gene therapy, wound healing, cell adhesion, guided bone regeneration, dental care, ophthalmic formulations, biological signaling, and as protein carriers, biocides, and delivery agents [40,41,42,43,44,45,46,47]. GG is used in various drug formulations like controlled release, continuous release, injectable nanoparticles, gel beads, and in situ gels [48,49,50,51]. A new class of GG with improved mechanical properties are prepared using methacrylation procedures [52]. GG is an US food and drug administration (FDA) & European union (EU) [53] approved biomaterial [45,54]. The mechanical properties of GG can be improved by modifying the type and the degree of crosslinks [52]. Cations can be used for cross linking GG [55], and covalent cross linking of GG gels improve its stability [56]. Commonly employed chemical cross linking, such as glycidyl methacrylation and methacrylic anhydride, can also be used to improve GG mechanical properties [56,57,58]. Modification of GG with tunable physical and mechanical properties have also been reported [52,59,60]. Physical and chemical crosslinking methods were studied for many natural materials, like hyaluronic acid (HA), alginate, gelatin, etc. [61,62,63,64]. The different strategies for preparing GG based materials are given in Figure 2.

## 2. Gellan Gum in Drug Delivery

GG based nano-hydrogel systems for multiple drug delivery applications were recently studied by many researchers. For example, prednisolone and paclitaxel were chemically linked to GG, and their anti-inflammatory and anti-cancer effects were studied in malignant cells [70]. A multi-particulate drug delivery system with many small units (0.05–2 nm) provide numerous advantages over a single unite system due to their smaller size. They are less dependent on gastric emptying, have increased bioavailability, cause less local irritation, and reduce the risk of systemic toxicity. They also have better reproducible pharmacokinetic behavior than conventional formulations, and better disintegration, even though they have some drawbacks [71]. They are formed by subunits, such as micro/macrobeads, granules, particles, pellets, spheres, and spheroids. Whereas, drug-loaded GG is prepared by a simple process, by external ionotropic gelation methods using a dropwise addition of aqueous GG with dissolved/dispersed drugs into aqueous solution of cations [72]. Several drugs, mainly antibiotics, were encapsulated with floating GG beads to increase their retention time in the stomach [72,73]. GG has advantageous properties over other existing materials, like being capable of contact with cations present in physiological fluids, mucoadhesiveness, nontoxicity, resistance to temperature, its biodegradability, persistence in the presence of the acid environment and enzymes in the gastrointestinal tract (GIT), stability, and high water holding capacity, etc. Due to these properties, it can be easily formulated into different forms, like particles, film, hydrogels, fibers, in situ gelling systems, and many other forms, with sustained and controlled drug release [74].

Gold nanoparticles (AuNPs) with controlled release and stabilized with GG, were studied in mouse embryonic fibroblast cells and human glioma cell lines, LN-229 and NIH 3T3 [75]. AuNPS stabilized by GG and with doxorubicin hydrochloride (DOX) were also studied for their drug release and cytotoxic effects, in human glioma stem cell lines HNGC-2 and LN 229 [76]. Antibacterial activity using silver nanoparticles (AgNPs) stabilized with GG, and their cytotoxic activity in mouse embryonic fibroblast cells (NIH 3T3), were also evaluated [77]. GG coated Gold nanorods (AuNRs) have also been prepared and studied by researchers [78], for intercellular drug delivery and imaging. Recent studies have shown that GG can be used in ocular, gastric, and nasal drug delivery applications [79,80]. Hydrocolloid bead based GG was studied for slow drug release applications [81]. GG was also used for protein delivery systems, including implant for insulin delivery in diabetic rats. The blood glucose levels of the implanted diabetic rats were reduced to half of those of blanks, and the therapeutic effects were found to last for a week [82]. Jeong et al. [83] used hesperidin (heteropolysaccharide), which is widely used in tissue engineering applications, along with GG for cartilage regeneration, and confirmed the cartilage regeneration, cell adhesion, and differentiation ability of the prepared scaffold, using 3-(4,5-Dimethylthiazol-2-yl)-2,5-Diphenyltetrazolium Bromide (MTT), SEM, and RT-PCR studies. In another study, Levofloxacin hemihydrate was used as an in situ gelling ophthalmic solution along with GG [84], researchers studied the in vitro gelation time, drug release and stability, absorbed gelling time (<15 s), and prolonged in vitro drug release (18–24 h), with a stability of 6 months at 25 °C/40 °C. Vashisth et al. [85] used ofloxacin loaded GG/polyvinyl alcohol (PVA) nanofibers for gastroretentive/mucoadhesive drug delivery applications, and their results showed a biphasic drug release pattern with considerable mucoadhesion and gastric retention, in rat gastric mucosal membranes. In another study by Vashisth et al. [86], they evaluated the GG/PVA nanofiber scaffold for skin tissue regeneration applications. They were characterized by SEM, the infrared spectra (IR), differential scanning calorimetry (DSC), and X-ray diffraction (XRD) analysis. Their biocompatibility and cell adhesion studies were confirmed by culturing with human dermal fibroblast (3T3L1) cells. A recent study, using resveratrol loaded chitosan/GG nanofibers as a novel gastrointestinal delivery system [87], highlighted that the encapsulation efficacy of resveratrol was 86 ± 6%. Antioxidant activities of resveratrol loaded nanofiber material were significantly higher than controls, and based on these findings, the authors suggested that prepared GG/chitosan resveratrol loaded nanofibers hold great potential as a drug delivery carrier. A study by Mehnath et al. [88] used Sericin-chitosan doped maleate GG nanocomposites for the maximum reduction and cellular damage of mycobacteria in *Mycobacterium tuberculosis* (TB) infections, this paved the way for the development of macromolecules in the pulmonary delivery of TB drugs. A detailed application of GG in drug delivery applications has been extensively reported for several applications (Table 1), including oral drug delivery formulations based on GG, ophthalmic drug delivery formulations, nasal drug delivery formulations, and topical drug delivery formulations [74,89].

## 3. Gellan Gum Cell Adhesion Properties

GG sponge-like hydrogels were reported to show optimal conditions for tissue engineering and regenerative medicine (TERM), due to their microstructure pore arrangement, mechanical stability, and high water content, which all together assist in cell adhesion and proliferation [32,67]. The GG cell adhesion properties are further improved by functionalizing them with bioactive peptide or protein conjugates. In the biomedical fields of TE and RM, cell adhesion and migration are vital to attain better results, those features are not demonstrated naturally by GG hydrogels. These features are improved by combining proteins and peptide sequences. GG microspheres were covalently functionalized with gelatin by Wang et al. [120], and partially denatured collagen derivatives through redox-mediated cross-linking, to enable anchorage-dependent cells (ADC) bindings. Human fetal osteoblasts and human dermal fibroblasts used in their study were successfully well adhered to the surface of the spheres. The good morphology, cell viability, and proliferation were observed in both cell lines. Silva et al. [125,145] proposed another strategy to improve cell adhesion on GG hydrogels. They modified GG using Diels–Alder clicks chemistry with synthetic peptide (GRGDS) derived from fibronectin. There are different studies reported previously that show the influence of hydrogel microstructure, like hydrophilicity and charge [146], degree of porosity and pore architecture [147], and matrix stiffness [148] on cell adhesion. GG combined with arginine-glycine-aspartic acid (RGD) sequences were reported to enhance integrin-mediated cell attachment [120].

**Figure 3:**
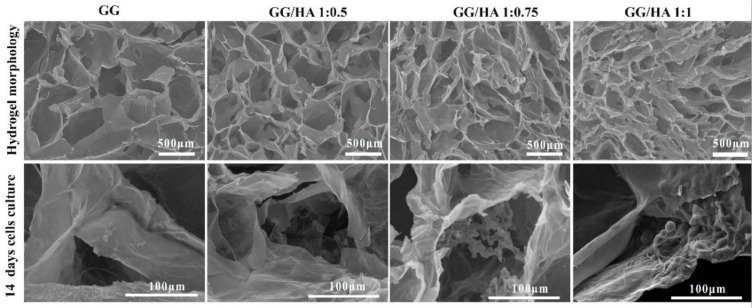
SEM observation of hydrogel morphology, and 14 days of culturing chondrocyte cells on the scaffold surface [35].

**Figure 4:**
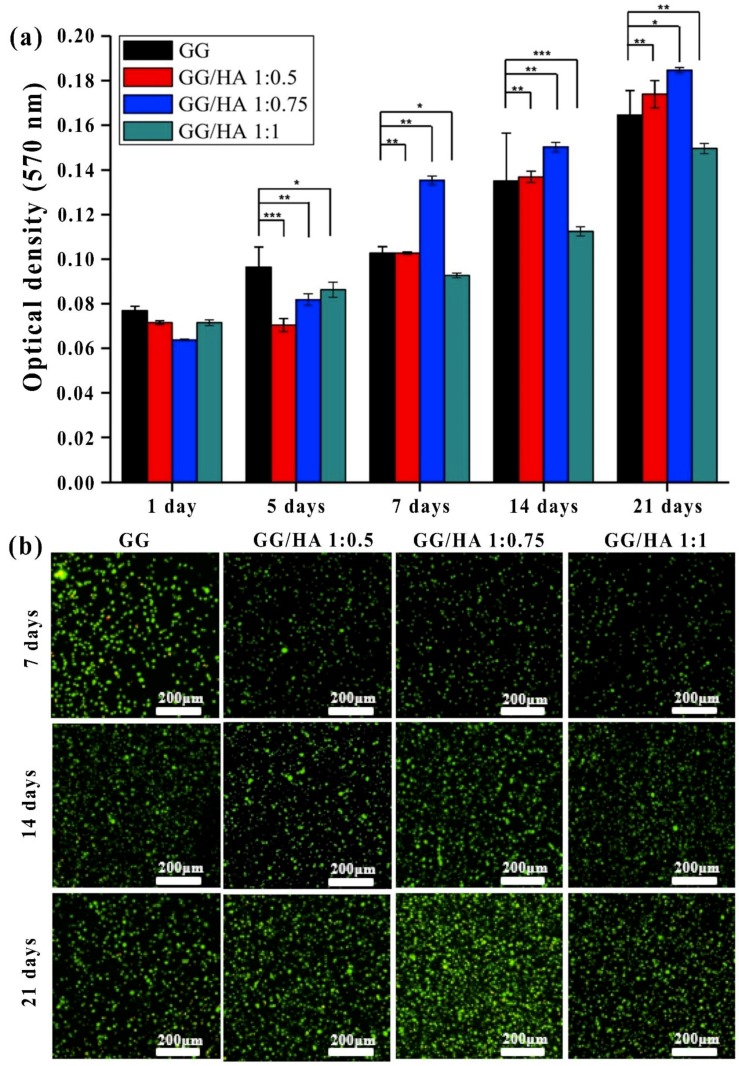
MTT assay (**a**) (values are mean ±  SD, n = 3, *p* < 0.05 (*), *p*  < 0.01 (**), and *p*  < 0.001 (***)), and live (green) and dead (red) images of cells encapsulated in hydrogel analyzed in z-stack mode (**b**) (scale bar = 100 μm) [35].

Kim et al. [35] prepared chondrocyte encapsulated GG-based HA blended hydrogel for cartilage regeneration, and they reported that the hydrogel enhanced cell adhesion, viability, proliferation, and gene expression in an in vitro and in an in vivo model. The microstructure and morphology of the hydrogels are provided in Figure 3. The cross-section images of normal cells and cells cultured on the hydrogels show the adhesion of cells on the surface. The pores are formed due to the ice crystal formation from freeze-drying steps, which were smaller when HA content was increased in the hydrogels. Compared with controls, of the other treated groups, the GG/HA 1:0.75 group showed a large amount of chondrocyte cells adhered on the surface which was supported by MTT assay results, and confocal fluorescent images performed at 7, 14 and 21 days (Figure 4)

**Figure 5:**
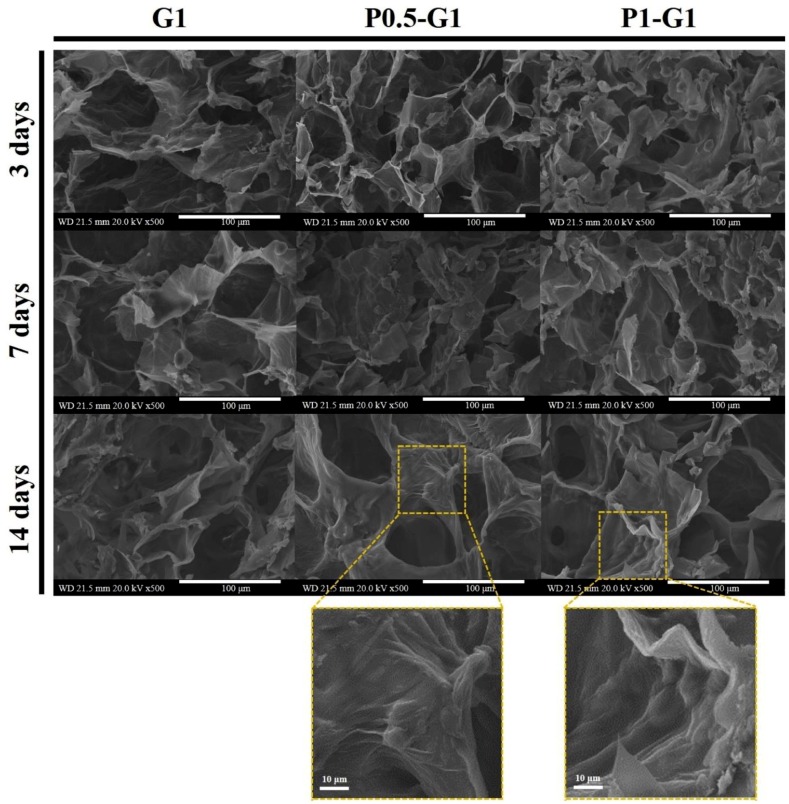
Scanning electron microscopic (SEM) images showing the cell adhesion and distribution on the surface of prepared hydrogels at 3, 7 and 14 days of culturing. G-gellan gum, P-poloxamer-heparin. The magnified images show the cells adhered to the hydrogel [65].

They studied the gene expression of collagen I and II in an in vitro model. In another study, GG/poloxamer-heparin (PoH) hydrogel was used as a carrier for bone marrow stem cell (BMSCs) delivery, in that experiment, they prepared a double network of GG hydrogel composed of PoH as a matrix platform for stem cell cultivation. They found that the hydrogel supported cell adhesion, distribution (Figure 5), and ECM production in an in vivo model [65]. Shin et al. [38] used GG/Silk fibroin (SF) for the chondrogenic differentiation ability of BMSCs, using miR-30.

**Figure 6:**
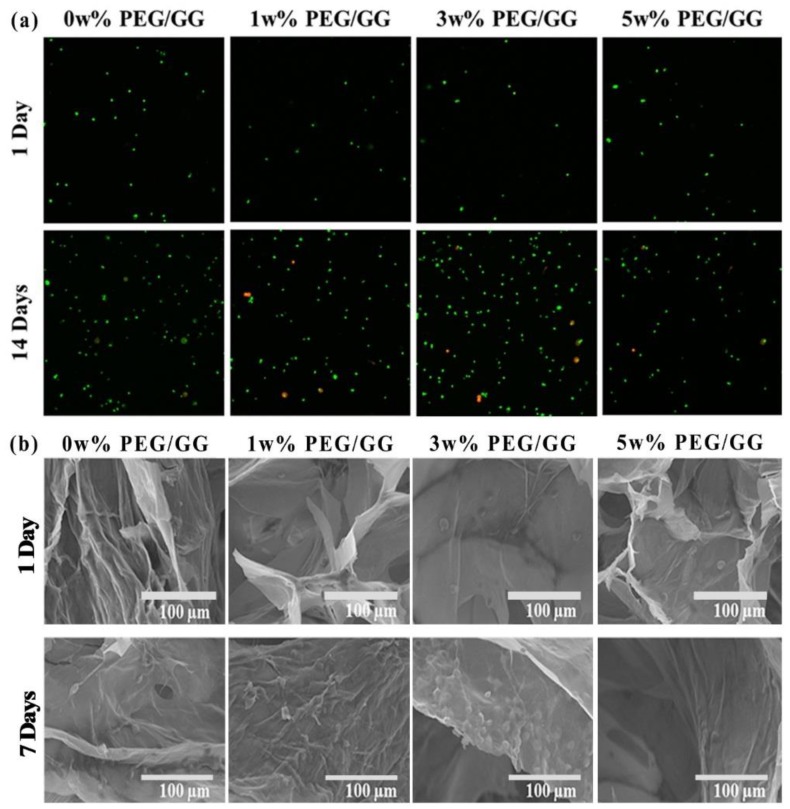
(**a**) Viability of human Retinal pigment epithelium (ARPE) live and dead cell staining images using a confocal Z-stack (100 μm), on PEG/GG hydrogels on days 1 and 14. Live and dead cells were stained in green and red, respectively. (**b**) SEM images showing the cell adhesion and proliferation on the surface of the hydrogels (PEG/GG) on days 1 and 7 [37].

Kim et al. [37] used GG/PEG hydrogel for engineering retinal pigment epithelial cells (RPECs) for transplantation. They prepared PEG/GG with different wt%, and RPECs were cultured on their surface to confirm their cell adhesion and growth properties. They observed biocompatibility (>90%) in the prepared hydrogels that were confirmed by confocal, scanning electron microscopy (SEM) and RT-PCR (Figure 6).

**Figure 7:**
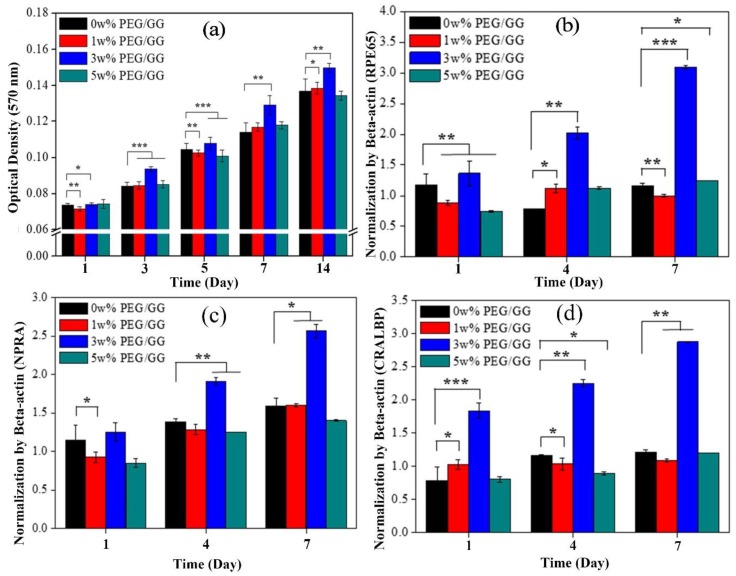
(**a**) Proliferation of ARPE at days 1, 3, 5, 7 and 14, studied by MTT assay (n = 3 in each group, p* < 0.5, p** <0.05, and p*** < 0.01). Gene expression of ARPE on PEG/GG hydrogels was analyzed by RT-PCR after 1, 4 and 7 days. (**b**) Quantitative analysis of retinal pigment epithelial 65 (RPE65) expression (**c**), Quantitative analysis of NPRA (**d**), Quantitative analysis of CRALBP normalized to Beta-actin (p* < 0.5, p** < 0.05, and p*** < 0.001) [37].

Confocal images showed significantly higher cell numbers at 14 days in the 3 w% PEG/GG hydrogel group, compared with other groups studied, including control groups. This was further confirmed by SEM images taken on days 1 and 7 of RPECs cultured on the surface of the hydrogel. The Proliferation of RPE cells was further confirmed by MTT, and gene expression studies using RT-PCR (Figure 7). The gene expression was normalized by β-actin, the gene expressions of RPE 65 (isomerase enzyme in RPE cells that catalyze a crucial step in the visual (retinoid) cycle), CRALBP (36-kDa water-soluble protein found in the retina and pineal gland that binds 11-*cis* retinol), and NPRA (which regulates the gene expression associated with RPE cell proliferation and sub-retinal fluid absorption) [149,150,151] were reported to be higher in the 3 w% hydrogel in all groups on all days, compared with other treated and control groups.

**Figure 8:**
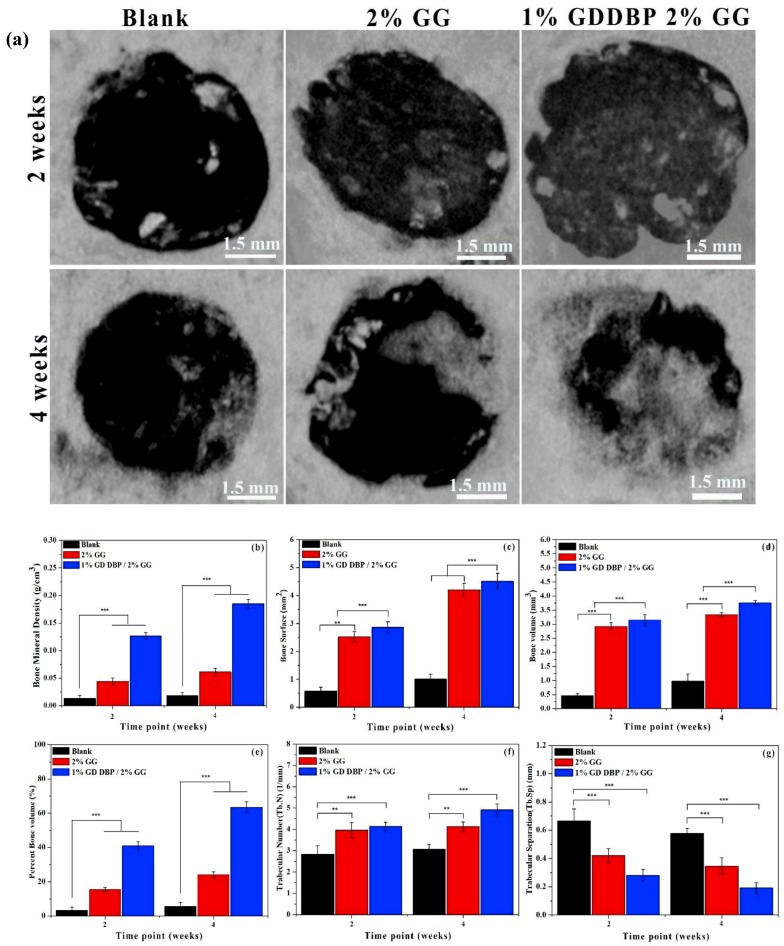
(**a**) Micro-CT images after in vivo implanted for 2 and 4 weeks, samples of blank, and 2% GG and 1% *Gallus gallus var domesticus* (GD) demineralized bone powder (DBP) 2% GG samples. (**b**) Bone mineral density (BMD), (**c**) Bone surface (BS), (**d**) Bone volume (BV), (**e**) Percent bone volume (PBV), (**f**) Trabecular number (Tb.N), and (**g**) Trabecular separation (Tb.Sp) (p* < 0.05, p** < 0.01, p*** < 0.001) [36].

In another study, Kim et al. [36] studied the application of a GG/demineralized bone powder (DBP) scaffold for bone tissue engineering applications in an in vitro and an in vivo model. Initial characterization was performed for the attachment and proliferation of BMSCs, after confirming their viability and their rate of proliferation, they were studied in an in vivo rat model and it was confirmed that the 1% GG/DBP showed better osteogenic effects, using micro CT analysis (Figure 8) and histology data. The quantitative evaluation of bone formation was measured using micro-CT, the results showed bone mineral density (BMD), bone surface (BS), bone volume (BV), Total volume (TV), BV/TV (Bone Volume over Total Volume), trabecular number (Tb.N), and trabecular separation (Tb.Sp), respectively. These factors play an important role in measuring bone regeneration and bone strength. The bone mineral density was reported to be significantly increased in treated groups, over a period of 4 weeks. Their findings clearly confirmed that the 1% GG/DBP scaffold significantly increased bone density in treated groups, compared with control and other groups.

**Figure 9:**
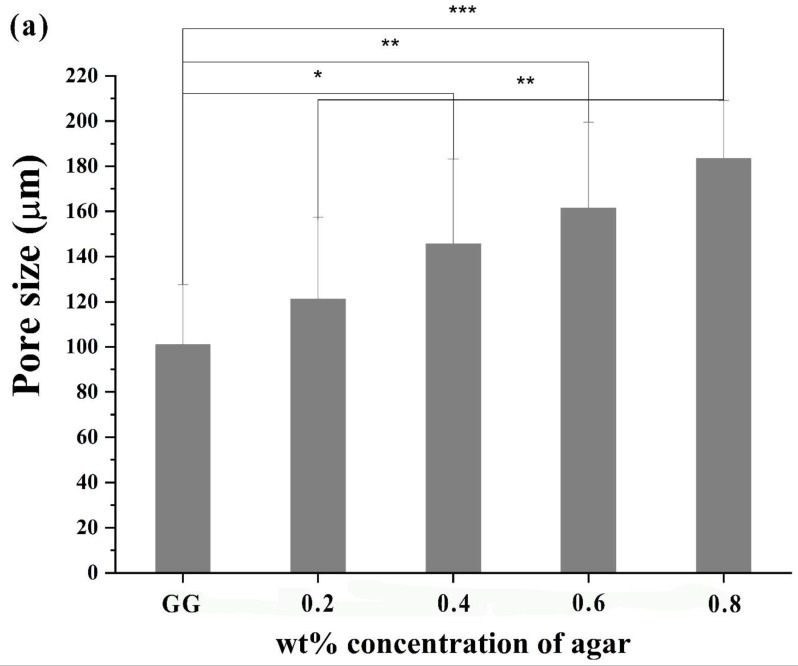

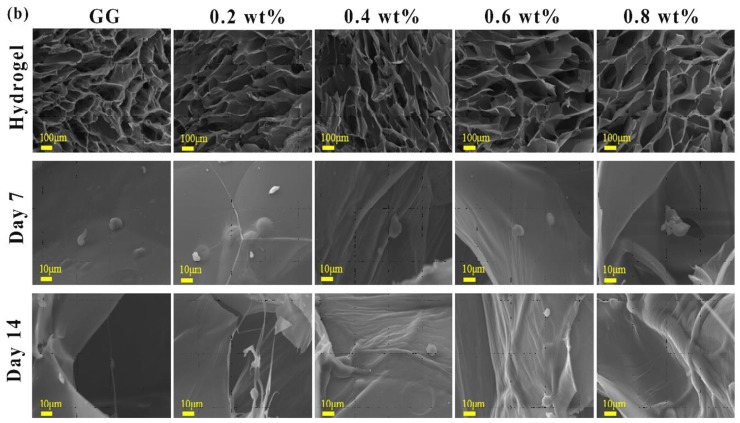
(**a**) Pore size of the hydrogels with different agar wt% (p* < 0.05, p** < 0.01, and p*** < 0.001). (**b**) SEM images of pristine hydrogel and chondrocytes morphology in GG and GG/Agar hydrogel, cultured for 7 and 14 days [39].

A recent study by Baek et al. [39] using GG/Agar, reported that the material demonstrated adhesion and proliferation of chondrocytes increased with the addition of agar. All the hydrogels were prepared using GG/Agar showed good cell adhesion and proliferation of rabbit chondrocytes. They also reported that the increasing agar concentration increased the pore size of the hydrogel and helped increase cell adhesion. The SEM image clearly represents cells seeded on the scaffolds present in a more spindle fibroblast-like shape, indicating an enhanced cell spreading with the synthesis of extracellular matrix. An increased amount of cell adhesion and proliferation were observed on the scaffold on days 7 and 14 (Figure 9). They also reported that the morphology of the material showed a suitable porous microstructure (between 70 and 180 µm), which allows for ideal water uptake for both increased mechanical properties, and optimal nutrient and oxygen diffusion for cells during growth.

## 4. Conclusion

This review informs readers about the applications of GG, particularly its emerging potential as a biomaterial for tissue engineering, drug delivery, cartilage regeneration, pharmaceutical, and ocular applications. However, the properties and extensive capacities of GG, such as biocompatibility, biodegradability, rapid gelation, water holding capacity, and non-toxicity, provides more opportunities for modifying, optimizing, and preparing many composites as biocompatible, hydrogels, scaffolds, porous material, drug delivery vehicles, cell carriers, and as active material in the pharmaceutical field, etc. As part of this review, we have collected and presented important research being carried out using GG alone, and in combination with other materials. The studies carried out with GG composites for several applications listed here are smaller, due to its diverse properties. The research being carried out by several researchers will surely bring many more advanced products, which will be beneficial for human beings in the future.

## Figures and Tables

**Table 1:** Gellan gum (GG) composites used in the biological fields for various applications.

Sl No	GG Composites	Applications	Reference
1	Xanthan gum (XG) -HAp	Bone tissue engineering	[90]
2	GG-XG-hyaluronan	Bone tissue engineering	[91]
3	GG/Starch	Drug delivery system	[92]
4	GG/alpha amylase	Pharmaceutical and biomedical	[5]
5	GG/PVA-Ofloxacin	Gastroretentive/mucoadhesive drug delivery	[85]
6	GG/kappa-carrageenan	Drugs on the ocular surface	[93]
7	GG/Chitosan	Nasal insert, antifungal agent, coatings, wound healing, antibiotic	[47,94,95,96] [14]
8	GG/kappa-carrageenan/alginates	Antifungal and antimicrobial drugs	[97]
9	GG/XG	Anti-adhesive	[98]
10	GG/pectin	Drug delivery	[15]
11	GG/agar	Biomedical applications	[99]
12	GG methacrylate/gelatin methacrylamide	In scaffolds for load-bearing tissues	[91]
13	GG/alginate	Sustained drug release	[16]
14	GG/titanium dioxide nanoparticles	Wound healing	[100]
15	GG/HAp	Bone, vasculature	[31,101] [102]
16	GG/Gelatin/genipin	material	[103]
17	GG/PLGA microsphere	Vertebra	[101]
18	GG/Gold nanorods	Bone	[104]
19	GG/Bioglass	Bone	[32,105]
20	GG/Graphine oxide	Scaffold	[106]
21	GG/HAGG/LAGG blends methacrylation/HA	Intervertebral discs	[106]
22	GG//methacrylation/GG microsphere/gelatin	Load bearing tissue	[107]
23	GG/methacrylation	Intervertebral discs, TE, cartilage repair	[58,108,109]
24	GG/Cinnamate	Wound healing	[110]
25	GG/Methacrylated gelatin	Cartilage	[111]
26	GG/HA	Skin repair/vascularization/cartilage regeneration	[102] [35]
27	GG/Laponite beads	Drug release	[112]
28	GG/ gum cordia	Drug delivery	[99]
29	GG/apigenin	Drug release	[113]
30	GG/avidin/boptinylated adhesive	Cell culture	[114]
31	GG/HAp/Lactoferrin	Bone tissue engineering	[115,116]
32	GG/AuNPs	Anti-cancer drug delivery	[75]
33	GG/AuNPs/DOX	Anti-cancer drug delivery	[76]
34	GG/AgNPs	Antibacterial, cytotoxic	[77]
35	GG/AuNRs	Intercellular drug delivery and imaging	[78]
36	GG/poloxamer 407/carbopol 934P)	Controlled delivery and antibacterial activity	[117]
37	GG/Lactoferrin	Bone Tissue Engineering	[115]
38	GG/insulin	Drug delivery	[82]
39	GG/poly(vinyl) alcohol	Tissue Engineering	[118]
40	GG/levofloxacin hemihydrate	Ophthalmic solution	[84]
41	GG/Polyvinylpyrrolidone (PVP)	Sustained release	[119]
42	Gelatin-grafted-GG-hydrogel microsphere	Cell encapsulation and delivery	[120]
43	GG hydrogel	Cartilage Tissue Engineering	[23], [121]
43	GG/fibrin/agarose	Cartilage regeneration	[122]
44	Ionic crosslinked methacrylated GG/Silk	Meniscus tissue engineering	[123]
45	GG/Polydopamine	Bone tissue engineering	[105]
46	GG/Collagen I, Beta -TCP	Bone graft material	[124]
47	GG-MA hydrogels	Intervertebral Disc	[28,106] [125]
48	GG/RGD	Cell adhesion, proliferation	[120]
49	GG/ UV crosslinked gelatin-methacryloyl (geMA)	Cartilage grafts bioprinting	[126,127]
50	GG/acrylamide grafted	Sustained release	[128]
51	GG/ dextran sulfates/ cellulose sulfate	Drug delivery	[129]
52	GG/polyvinylalcoho	Beta-blocker	[130]
53	GG/alginate	Antibiotic, Antinflammatory	[73,131]
54	GG/polyvinylalcohol	Antibiotic	[132]
55	GG/hyaluronic acid ester/polyvinylalcohol	Wound healing	[133]
56	GG/chitosan/PEG	Wound healing	[134]
57	GG/glucosamine	Oral cancer treatment	[135]
58	GG/ HA	Cartilage regeneration	[35]
59	GG/ poloxamer-heparin	Bone marrow stem cells delivery	[65]
60	GG/PEG	Retinal pigment epithelial cells regeneration	[37]
61	GG/ demineralized bone powder	Bone tissue regeneration	[36]
62	GG/Agar	Cartilage regeneration	[39]
63	GG/Silk fibroin	Chondrogenic differentiation	[38]
64	GG/Saponin	Cartilage regeneration	[40]
65	GG/Chondroitin sulfate	Cartilage regeneration	[41]
66	GG/ Gelatin	Cartilage regeneration	[136]
67	GG/Hesperidin	Cartilage regeneration	[83]
68	GG/ duck feet derived collagen	Tissue Engineering	[137]
69	GG hydrogel	Intervertebral disc	[106]
70	GG/ polyvinyl alcohol	Skin tissue regeneration	[86]
71	GG/PVA/Water	Skin tissue regeneration	[138]
72	GG/Chitosan/ resveratrol	Gastrointestinal delivery	[87]
73	GG/apigenin	Oral drug delivery	[113]
74	GG/Laponite Beads	Gastrointestinal drug release	[112]
75	Maleate GG/Sericin-chitosan	*Mycobacterium tuberculosis*	[88]
76	GG/sodium alginate/low-methoxyl pectin coated carboxymethyl pullulan-ZnO nanocomposites encapsulating erlotinib	Lung cancer therapy	[139]
77	GG/Triamcinolone acetonide	Topical Ocular Delivery	[140]
78	GG/Sericin/rice bran albumin	Cancer drug delivery	[141]
79	GG/natamycin bilosomes	Ocular pharmacotherapy	[142]
80	GG/Methotrexate	Drug delivery	[143]
81	GG/brinzolamide	Ocular delivery	[144]
